# Efficacy of tetracyclines and fluoroquinolones for the treatment of macrolide-refractory *Mycoplasma pneumoniae* pneumonia in children: a systematic review and meta-analysis

**DOI:** 10.1186/s12879-021-06508-7

**Published:** 2021-09-25

**Authors:** Jong Gyun Ahn, Hye-Kyung Cho, Donghe Li, Miyoung Choi, Jina Lee, Byung-Wook Eun, Dae Sun Jo, Su Eun Park, Eun Hwa Choi, Hyeon-Jong Yang, Ki Hwan Kim

**Affiliations:** 1 Department of Pediatrics, Severance Children’s Hospital, Yonsei University College of Medicine, Seoul, Korea; 2 Institute for Immunology and Immunological Diseases, Yonsei University College of Medicine, Seoul, Korea; 3 Department of Pediatrics, Gachon University College of Medicine, Gil Medical Center, Incheon, Korea; 4 Interdisciplinary Program of Bioinformatics, Seoul National University, Seoul, Korea; 5 Division of Health Technology Assessment Research, National Evidence-Based Healthcare Collaborating Agency (NECA), Seoul, Korea; 6 Department of Pediatrics, University of Ulsan College of Medicine, Ulsan, Korea; 7 Department of Pediatrics, School of Medicine, Eulji University, Daejeon, Korea; 8 Department of Pediatrics, Chonbuk National University Medical School, Jeonju, Korea; 9 Department of Pediatrics, School of Medicine, Pusan National University, Pusan, Korea; 10 Department of Pediatrics, Seoul National University College of Medicine, Seoul, Korea; 11 Pediatric Allergy and Respiratory Center, Department of Pediatrics, SCH Biomedical Informatics Research Unit, Soonchunhyang University Seoul Hospital, Soonchunhyang University College of Medicine, Seoul, Korea; 12 Department of Pediatrics, College of Medicine, The Catholic University of Korea, Seoul, Korea; 13 Incheon St. Mary’s Hospital, The Catholic University of Korea College of Medicine, 59 Dongsu-ro, Bupyeong-gu, Incheon, Korea

**Keywords:** *Mycoplasma pneumoniae*, Macrolide-resistant, Tetracycline, Fluoroquinolone, Child

## Abstract

**Background:**

*Mycoplasma pneumoniae* is a common pathogen that causes community-acquired pneumonia in school-age children. Macrolides are considered a first-line treatment for *M. pneumoniae* infection in children, but macrolide-refractory *M. pneumoniae* (MRMP) strains have become more common. In this study, we assessed the efficacy of tetracyclines and fluoroquinolones in MRMP treatment in children through a systematic review and meta-analysis.

**Methods:**

Two reviewers individually searched 10 electronic databases (Medline/Pubmed, Embase, the Cochrane Library, and core Korean, Chinese, and Japanese journals) for papers published from January 1, 1990 to March 8, 2018. The following data for each treatment group were extracted from the selected studies: intervention (tetracyclines and fluoroquinolones/comparator), patient characteristics (age and sex), and outcomes (fever duration, hospital stay length, treatment success rate, and defervescence rates 24, 48, and 72 h after starting treatment).

**Results:**

Eight studies involving 537 participants were included. Fever duration and hospital stay length were shorter in the tetracycline group than in the macrolide group (weighted mean difference [WMD] = − 1.45, 95% confidence interval [CI]: − 2.55 to − 0.36, *P* = 0.009; and WMD = − 3.33, 95% CI: − 4.32 to − 2.35, *P* < 0.00001, respectively). The therapeutic efficacy was significantly higher in the tetracycline group than in the macrolide group (odds ratio [OR]: 8.80, 95% CI: 3.12–24.82). With regard to defervescence rate, patients in the tetracycline group showed significant improvement compared to those in the macrolide group (defervescence rate after 24 h, OR: 5.34, 95% CI: 1.81–15.75; after 48 h, OR 18.37, 95% CI: 8.87–38.03; and after 72 h, OR: 40.77, 95% CI: 6.15–270.12). There were no differences in fever improvement within 24 h in patients in the fluoroquinolone group compared to those in the macrolide group (OR: 1.11, 95% CI: 0.25–5.00), although the defervescence rate was higher after 48 h in the fluoroquinolone group (OR: 2.78, 95% CI: 1.41–5.51).

**Conclusion:**

Tetracyclines may shorten fever duration and hospital stay length in patients with MRMP infection. Fluoroquinolones may achieve defervescence within 48 h in patients with MRMP infection. However, these results should be carefully interpreted as only a small number of studies were included, and they were heterogeneous.

**Supplementary Information:**

The online version contains supplementary material available at 10.1186/s12879-021-06508-7.

## Background

*Mycoplasma pneumoniae* (MP) is a common causative pathogen of community-acquired pneumonia (CAP) worldwide, particularly in school-age children and adolescents [1, 2]. The prevalence of pediatric CAP ranges from 10 to 40% [3]. Although MP infection often causes self-limiting disease, it may also develop into severe pneumonia with extra-pulmonary complications [4, 5].

β-Lactam antibiotics, which are active against most respiratory bacterial pathogens, are ineffective against MP due to the lack of cell wall. Protein synthesis inhibitors, such as macrolides and tetracyclines, or DNA synthesis inhibitors, such as fluoroquinolones, are usually effective against MP in vitro, and are the drugs of choice for MP infections. In children, macrolides are the only recommended first-line treatment for MP infection due to age-related safety issues with the use of tetracyclines and fluoroquinolones [6]. However, in recent years, the prevalence of macrolide-refractory MP (MRMP) infection has rapidly increased among children, particularly in East Asian countries such as Korea, Japan, and China [7–9].

The clinical implications of MRMP have not been fully elucidated with regard to whether resistant strains can cause more serious or long-term disease, and macrolides may be clinically effective even in the presence of resistance [10, 11]. However, several studies have revealed that MRMP is associated with a longer febrile and hospital stay period, prolonged antibiotic use, and a high frequency of pneumonia aggravation and extrapulmonary complications [10]. Therefore, alternative antibiotic treatment options are needed in severe MRMP cases with clinical deterioration.

Alternative antibiotic treatment options for MRMP infections include tetracyclines and fluoroquinolones. However, the use of these agents in children is limited because of their toxicity. Tetracyclines may cause adverse effects such as hypoplasia of the enamel, permanent gray/brown staining of the teeth, and transient anostosis in children [12]. Therefore, they are contraindicated for patients younger than 8 years. Fluoroquinolones are not usually prescribed as a first-line therapy for CAP in children as they have been reported to cause cartilage erosion in young animals [13]. Despite this concern, fluoroquinolones have been safely used to treat severe infections in children in the absence of other safe and effective alternatives [14].

Recently, studies have reported the use of second-line antimicrobial agents such as tetracyclines and fluoroquinolones for treating MRMP infection in children. In this study, we evaluated the efficacy of tetracyclines and fluoroquinolones against MRMP infection in children through a systematic review and meta-analysis.

## Methods

### Search strategy

To identify relevant studies, we performed an extensive search across 10 electronic full-text databases [Medline/Pubmed, Embase, the Cochrane Library, KoreaMed (https://koreamed.org), National Digital Science Library (http://www.ndsl.kr), Korean medical database (http://kmbase.medric.or.kr), Research Information Sharing Service (http://www.riss.kr), Koreanstudies Information Service System (http://kiss.kstudy.com), China National Knowledge Infrastructure (http://www.cnki.net), and Japan Medical Abstracts Society, Igaku Chuo Zasshi (http://www.jamas.or.jp)] with no language restrictions. Two independent medical librarians (D.W.S. and M.L.) searched articles published from January 1, 1990 to March 8, 2018 using a protocol designed for this study. The search terms used for each database are listed in Additional file 1. Databases from Korea, China, and Japan were chosen as the data from these have shown a high prevalence of MP and MRMP.

### Eligibility

Articles that met the following inclusion criteria were included: (1) the study topic was MRMP, defined as disease showing no clinical or radiological improvement 48–72 h after macrolide administration; (2) the subjects were children aged ≤ 18 years; (3) the study was designed as a randomized controlled trial (RCT) or an observational study with controls; (4) the intervention agent was a non-macrolide antibiotic known to be active against MP, such as tetracyclines and fluoroquinolones; (5) the control was a macrolide drug; and (6) at least one of the predetermined outcomes was reported.

Animal and preclinical studies, as well as articles other than original research articles (e.g., reviews, editorials, letters, conference abstracts, and comments) were excluded. Studies with duplicate subjects (i.e., different studies using the same outcome indicators in the same number of patients) were also excluded. Our search strategy implemented no language restrictions, and non-English articles were translated and included for evaluation.

### Study selection, quality assessment, and data extraction

**Fig. 1:**
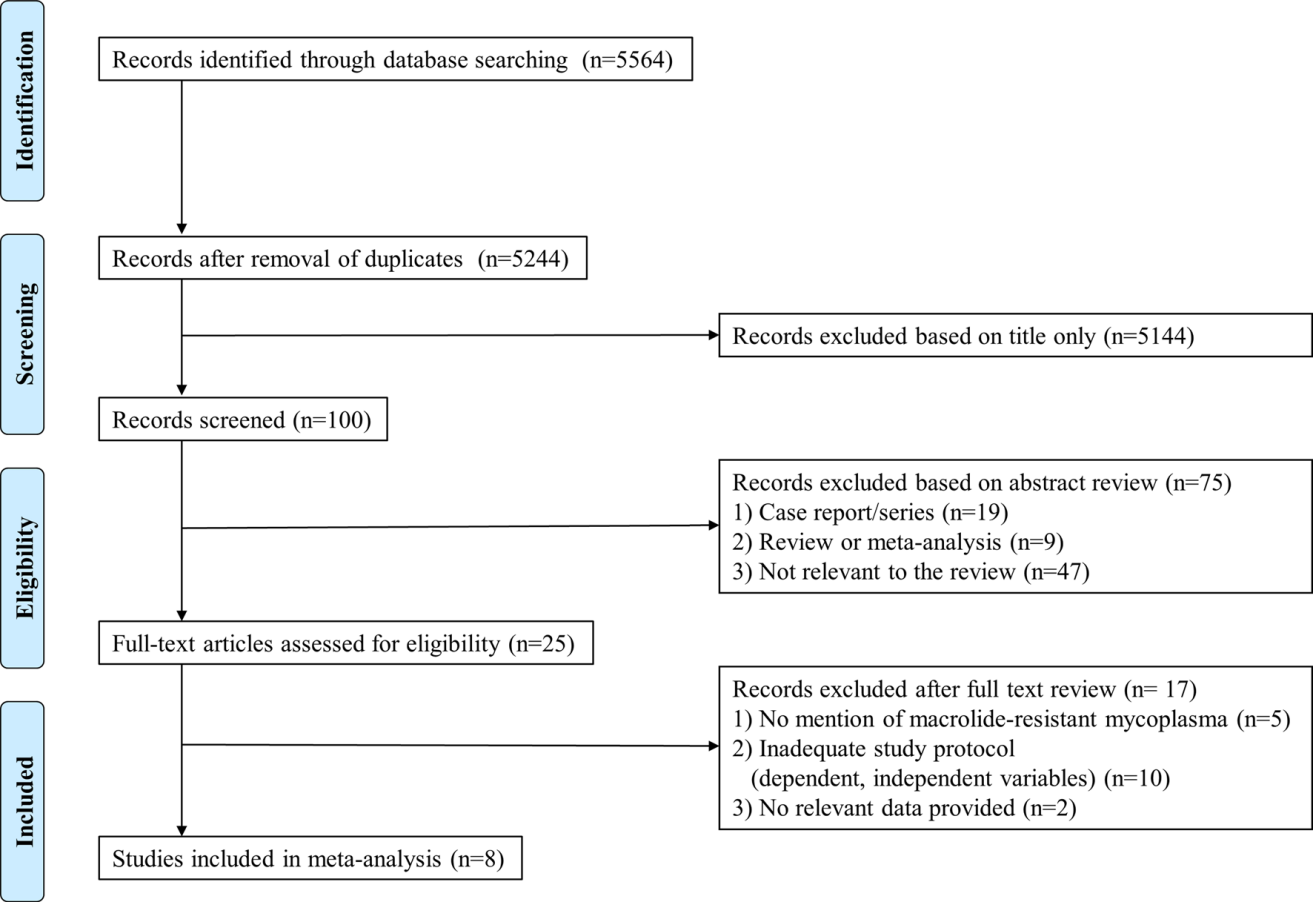
Flowchart of the selection process of studies included in the meta-analysis

Studies were initially screened by two independent reviewers (J.G.A. and H.K.C.) based on the title and abstract, followed by full-text screening. The literature selection process was conducted in accordance with the Preferred Reporting Items for Systematic Reviews and Meta-Analysis Protocols 2015 statement (Fig. 1) [15]. The quality of the selected studies was assessed using the Cochrane Risk of Bias Tools [16] for RCTs and the revised Risk of Bias Tool for Non-Randomized Studies [17] for observational studies. The data extraction form included the following information: first author, year of publication, population in each group, antibiotic treatment (tetracycline or fluoroquinolone/comparator), patient characteristics (age and sex), and outcomes (durations of fever and hospitalization, therapeutic efficacy, and defervescence rates at 24, 48, and 72 h after starting treatment). Therapeutic efficacy was defined as the rate of achieving clinical recovery with no fever, improvement or disappearance of cough, and improved or normal laboratory values. Study selection, quality assessment, and data extraction were conducted by two independent reviewers (J.G.A. and H.K.C.). Any disagreements were resolved through discussion with a third reviewer (K.H.K.). If the results of the selected studies were unclear or missing, we contacted the corresponding study investigators to obtain or confirm data.

### Statistical analysis

We pooled the findings from the included studies and calculated mean, standard deviation, and sample size. For outcomes presented as continuous variables, such as fever duration, hospital stay length, and therapeutic efficacy, we calculated mean differences with 95% confidence intervals (CIs). For dichotomous outcomes such as the achievement of defervescence after 24, 48, and 72 h of treatment, we calculated odds ratios (ORs) with 95% CIs. The average effect summary was calculated using a random-effects model (Mantel–Haenszel method) using Review Manager 5.3 (The Cochrane Collaboration, London, UK) including the *I*^2^ statistic. *I*^2^ of 25%, 50%, and 75% indicated low, moderate, and high heterogeneity, respectively [18]. To assess the risk of publication bias, we used funnel plots for visual inspection; Egger test and the trim-and-fill method were used for statistical identification.

## Results

**Fig. 2:**
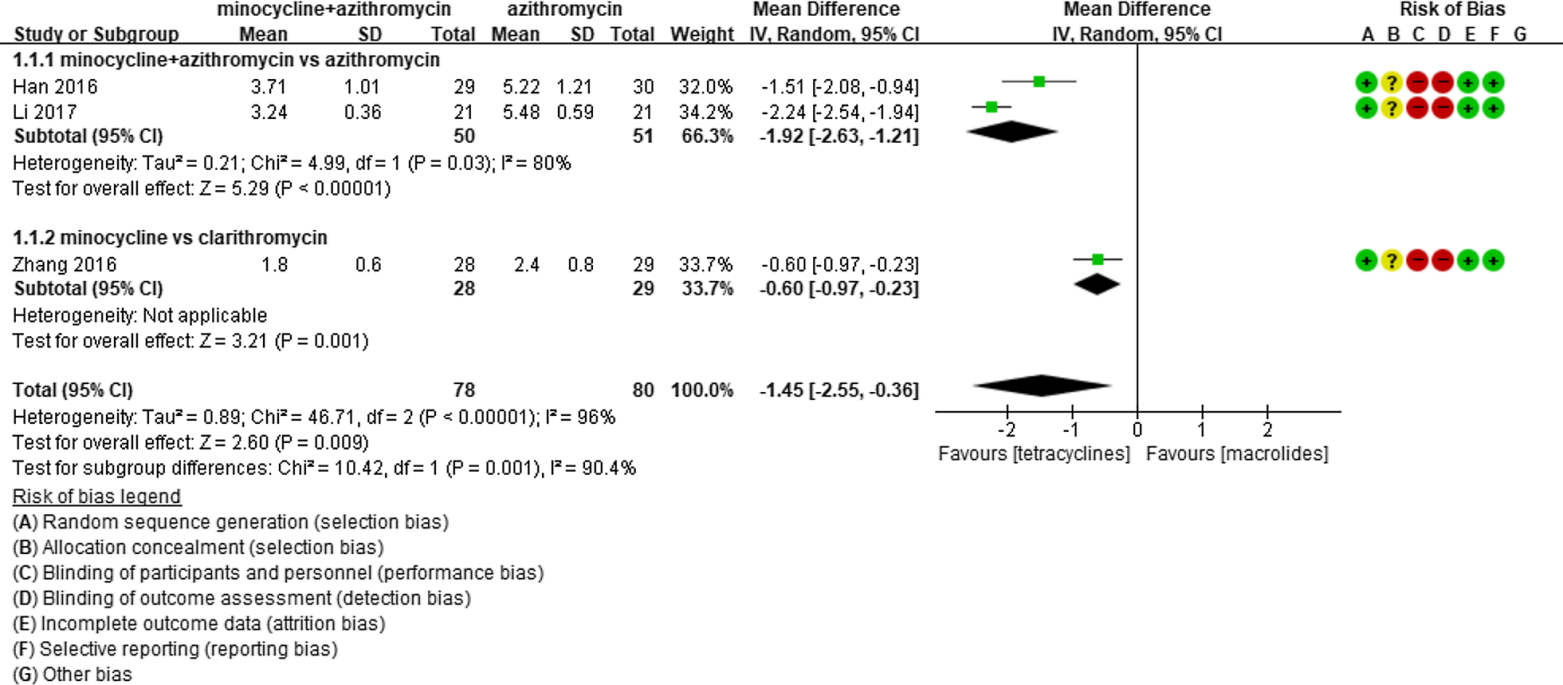
Comparison of fever duration between patients treated with tetracyclines and those treated with macrolides

**Fig. 3:**
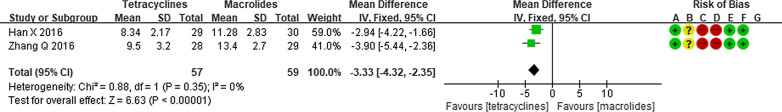
Comparison of hospital stay length between patients treated with tetracyclines and those treated with macrolides

**Fig. 4:**
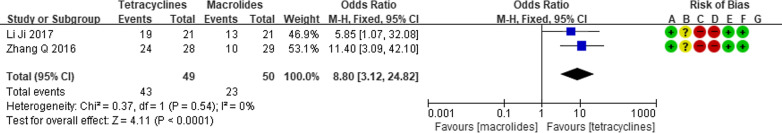
Comparison of treatment efficacy between patients treated with tetracyclines and those treated with macrolides

**Fig. 5:**
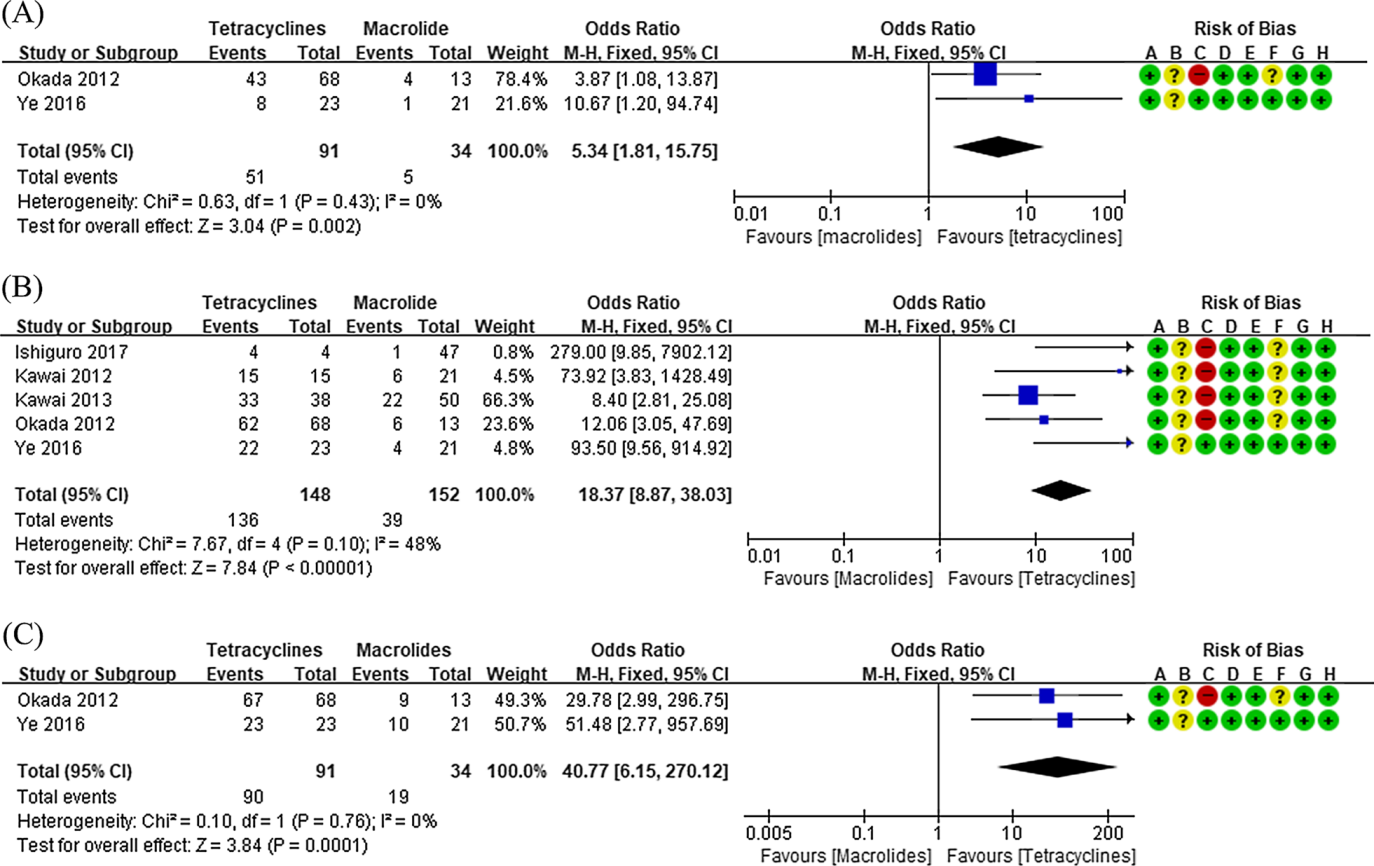
Forest plots of defervescence rates in patients treated with tetracyclines or macrolides. The defervescence rates at 24 h (**a**), 48 h (**b**), and 72 h (**c**) are shown

**Fig. 6:**
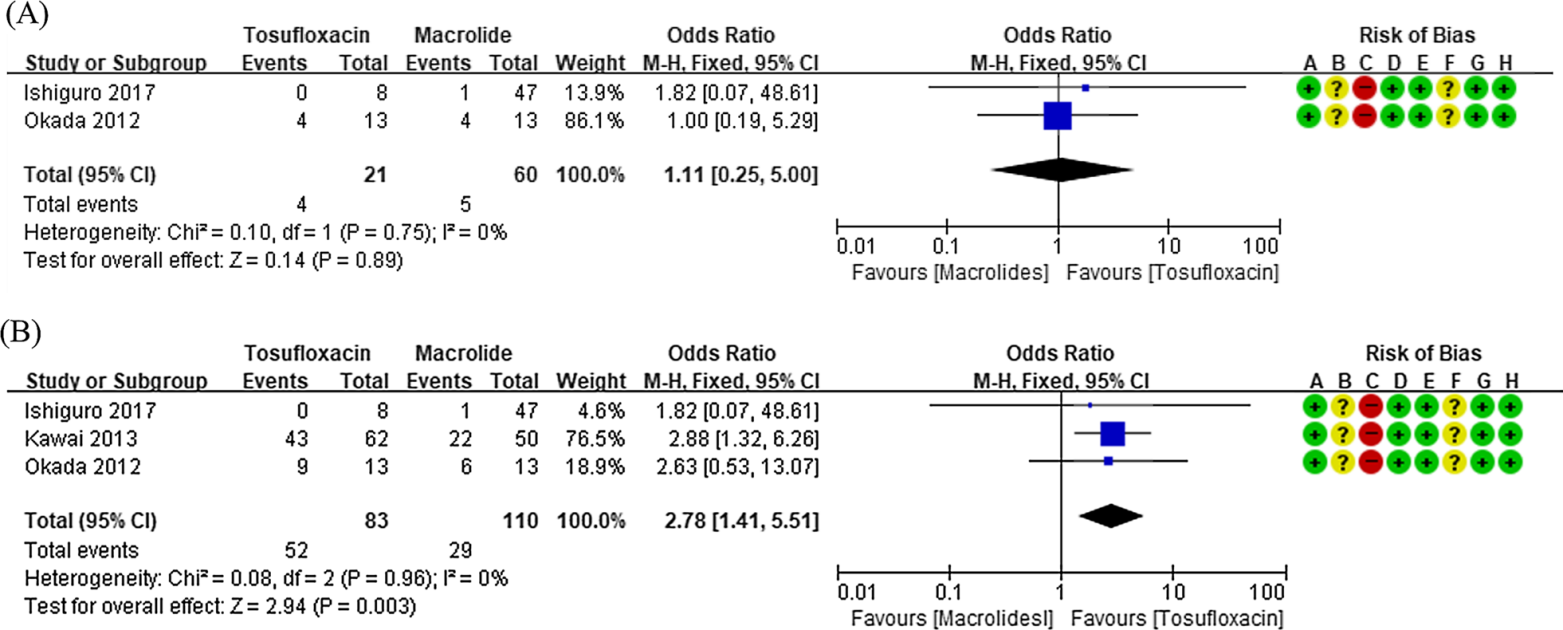
Forest plots of defervescence rates in patients treated with tosufloxacin or macrolides. The defervescence rates at 24 h (**a**) and 48 h (**b**) are shown

**Fig. 7:**
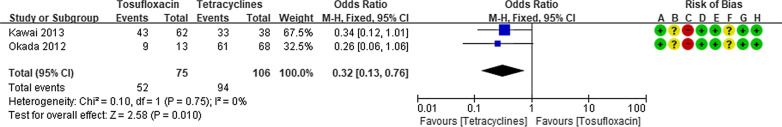
Forest plot of 48-h defervescence rates in patients treated with tosufloxacin or tetracyclines

Eight studies involving 537 participants were reviewed in this study. The characteristics of the studies included in this meta-analysis are presented in Table 1 and bias assessment results are shown in Figs. 2, 3, 4, 5, 6, 7. Publication bias was not assessed, because only a few trials were included and therefore, appropriate assessment with funnel plots or advanced regression-based methods could not be performed.

**Table 1:** Summary of studies included in the meta-analysis

Study author (year)	Country	Age^*^		Number of subjects		Treatment		Outcome measure analyzed
		Intervention	Control	Intervention	Control	Intervention	Control	
Han (2016) [19]	China	9.54 ± 1.20	10.32 ± 1.76	TET (n = 29)	Macrolide (n = 30)	AZM therapy same as the control, with the addition of MIN HCl 4 mg/kg once, followed by 2 mg/kg orally 24 h later and every 12 h for 5 days	AZM 10 mg/kg/d, intravenously for 5 days, and then discontinued for 3 days. If necessary, two courses were administered	Fever duration, hospital stay length
Li (2017) [20]	China	10.54 ± 0.38	10.61 ± 0.36	TET (n = 21)	Macrolide (n = 21)	AZM therapy same as the control, with the addition of MIN50 mg orally twice daily	AZM 10 mg/kg/day, intravenously for 5 days, and then discontinued for 3 days. If necessary, a second course was administered for 3 days	Fever duration, therapeutic efficacy
Zhang (2016) [21]	China	10.3 ± 1.7	10.1 ± 1.7	TET (n = 28)	Macrolide (n = 29)	MIN 100 mg orally twice daily for 2 weeks	CLR 250 mg three times daily for 2 weeks	Fever duration, length of hospital stay, therapeutic efficacy
Okada (2012) [22]	Japan	8 (1–14)		TET (n = 68) Fluoroquinolone (n = 13)	Macrolide (n = 13)	MIN 4 mg/kg/day twice daily; DOX 4 mg/kg/day twice daily; tosufloxacin 12 mg/kg/day twice daily	AZM, CLR	Defervescence after 24, 48, and 72 h
Ye (2016) [23]	China	9.2 ± 1.6	9.3 ± 1.0	TET (n = 23)	Macrolide (n = 21)	DOX 4 mg/kg/d	AZM 10 mg/kg/day	Defervescence after 24, 48, and 72 h
Kawai (2012) [24]	Japan	Unknown		TET (n = 15)	Macrolide (n = 21)	MIN 2–4 mg/kg/day	AZM 10 mg/kg/day; CLR 10–15 mg/kg/day	Defervescence after 48 h
Kawai (2013) [25]	Japan	8.0 (0–15)		TET (n = 38) Fluoroquinolone (n = 62)	Macrolide (n = 50)	MIN 4 mg/kg twice daily; tosufloxacin 12 mg/kg twice daily	AZM 10 mg/kg once daily; CLR 15 mg/kg twice daily	Defervescence after 48 h
Ishiguro (2017) [26]	Japan	9.0 ± 3.2		Fluoroquinolone (n = 8)	Macrolide (n = 47)	MIN 2–4 mg/kg/day for 2–4 days; tosufloxacin 12 mg/kg/day for 3–7 days	AZM 10 mg/kg/day for 3 days; CLR 10–15 mg/kg/day for 3–7 days	Defervescence after 24, 48, and 72 h

*AZM* azithromycin, *CLR* clarithromycin, *DOX* doxycycline, *MIN* minocycline, *TET* tetracycline

^*^Presented as mean ± standard deviation or median (range)

### Macrolides vs. tetracyclines

#### Fever duration

In the three RCTs included [19–21], fever duration was shorter in the tetracycline group than in the macrolide group (weighted mean difference [WMD] =  − 1.45, 95% CI: − 2.55 to − 0.36, *P* = 0.009). However, considering the significant inter-study heterogeneity (*I*^2^ > 50%), we conducted subgroup analysis to compare the effects of combination treatment with tetracycline and macrolide with that of tetracycline-only treatment. Subgroup analysis revealed the superior effects of tetracycline compared with macrolide. The high heterogeneity of the combination subgroup (tetracycline and macrolide treatment) can be attributed to the high effect observed in Li’s study. Therefore, these outcomes were assessed using a random-effects model considering inter- and intra-study variation, which confirmed that fever duration was significantly shorter in the tetracycline group than in the macrolide group (combination treatment vs. macrolide-only treatment, WMD = − 1.92, 95% CI: − 2.63 to − 1.21, *P* < 0.00001; tetracycline-only treatment vs. macrolide-only treatment, WMD = − 0.60, 95% CI: − 0.97 to − 0.23, *P* = 0.001, Fig. 2).

#### Hospital stay length

In two RCTs [19, 21], the length of hospital stay was shorter in the tetracycline group than in the macrolide group (WMD = − 3.33, 95% CI: − 4.32 to − 2.35, *P* < 0.00001, Fig. 3). There was no significant inter-study heterogeneity in terms of outcome (*I*^2^ < 50%).

#### Therapeutic efficacy

Two RCTs [20, 21] were assessed to compare treatment efficacy in the tetracycline and macrolide groups. Therapeutic efficacy was significantly higher in the tetracycline group than in the macrolide group (OR: 8.80, 95% CI: 3.12–24.82, Fig. 4). There was no significant inter-study heterogeneity in terms of outcome (*I*^2^ < 50%).

#### Defervescence after 24, 48, and 72 h

In the two prospective observational studies included [22, 23], the defervescence rate 24 h after starting treatment was compared between tetracycline- and macrolide-treated groups. The 24-h defervescence rate was significantly higher in the tetracycline group than in the macrolide group (OR: 5.34, 95% CI: 1.81–15.75, Fig. 5a). There was no significant inter-study heterogeneity in this outcome (*I*^2^ < 50%).

In the five prospective observational studies assessing the defervescence rate 48 h after starting treatment [22–26], the 48-h defervescence rate was higher in the tetracycline group than in the macrolide group (OR: 18.37, 95% CI: 8.87–38.03, Fig. 5b). There was no significant inter-study heterogeneity in terms of this outcome (*I*^2^ < 50%).

Two prospective observational studies [22, 23] compared the defervescence rate between the tetracycline and macrolide groups 72 h after starting treatment. The 72-h defervescence rate was higher in the tetracycline group than in the macrolide group (OR: 40.77, 95% CI: 6.15–270.12, Fig. 5c). There was no significant inter-study heterogeneity with regard to this outcome (*I*^2^ < 50%).

### Macrolides vs. tosufloxacin

#### Defervescence after 24 and 48 h

There was no significant difference between the tosufloxacin and macrolide groups with regard to 24-h defervescence rate in two prospective observational studies [22, 26] (OR: 1.11, 95% CI: 0.25–5.00, Fig. 6a). There was no significant inter-study heterogeneity with regard to this outcome (*I*^2^ < 50%).

Three prospective observational studies [22, 25, 26] compared the 48-h defervescence rate between the tosufloxacin and macrolide groups. The tosufloxacin group showed a higher defervescence rate than the macrolide group (OR: 2.78, 95% CI: 1.41–5.51, Fig. 6b). There was no significant inter-study heterogeneity with regard to this outcome (*I*^2^ < 50%).

### Tetracyclines vs. tosufloxacin

#### Defervescence after 48 h

Two prospective observational studies [22, 25] compared the defervescence rate 48 h after starting treatment between the tosufloxacin and tetracycline groups. The tetracycline group showed a significantly higher 48-h defervescence rate than the tosufloxacin group (OR: 0.32, 95% CI: 0.13–0.76, Fig. 7). There was no significant inter-study heterogeneity in terms of this outcome (*I*^2^ < 50%).

## Discussion

To the best of our knowledge, this is the first meta-analysis to assess the efficacy of tetracyclines and fluoroquinolones against MRMP infection in children. Although the clinical relevance of macrolide-resistant strains has not been established, severe refractory cases caused by such strains have been reported, and there is a need for alternative treatments [11]. Several studies on antibiotic treatment of MRMP infections have been published in the past decade, but the number of subjects has been small; moreover, multinational studies have not been conducted. Therefore, in this meta-analysis, data from multiple countries were combined to compare the effect of secondary antibiotics with that of macrolide treatment in MRMP treatment. In this systematic review and meta-analysis, we evaluated three RCTs and five prospective observational studies comparing treatment responses between the macrolide and second-line antibiotic treatment groups in children with MRMP infection. Our review revealed that tetracyclines can shorten fever duration and length of hospital stay, as well as achieving defervescence at 24, 48, and 72 h after starting treatment; with fluoroquinolones, defervescence can be achieved within 48 h. Although some data suggested that tetracyclines may be more effective than fluoroquinolones in achieving defervescence after 48 h in patients with MRMP infection, there was not enough evidence to determine the superiority of one group over the other.

Tetracyclines are recommended only for use in children aged ≥ 8 years based on reports of permanent tooth discoloration and tooth enamel hypoplasia in children receiving first-generation tetracyclines [12]. Previous studies have reported that visible dental staining occurred in 23–92% of children treated with tetracyclines [27–31], and it correlated with the dose and duration of treatment [12, 27]. However, data showing an association between treatment with new-generation tetracyclines, such as doxycycline and minocycline, and dental staining are limited. A recent study reported that the short-term use of doxycycline in children aged < 8 years for the treatment of Rocky Mountain spotted fever did not cause dental staining, enamel hypoplasia, or changes in tooth color [32]. In addition, updated recommendations from the American Academy of Pediatrics now include the use of doxycycline for ≤ 21 days in children of all ages, on the grounds that doxycycline binds less avidly to calcium than other tetracyclines and that the risk of dental staining associated with short courses is minimal [33]. All tetracyclines in studies included in our meta-analysis were new-generation tetracyclines (minocycline, n = 6; doxycycline, n = 1). Most of the patients in the tetracycline group were aged ≥ 8 years, and they benefited from this treatment. Considering that new-generation tetracyclines are licensed for use in patients aged ≥ 8 years in many countries, they may be the preferred choice for treating MRMP infections in children aged ≥ 8 years.

The use of fluoroquinolones in children is usually reserved for specific indications because of their potential risk of musculoskeletal toxicity. Currently, they are approved by the U.S. Food and Drug Administration for use in children aged < 18 years only for the treatment of complicated urinary tract infections and pyelonephritis and the treatment and prevention of inhalation anthrax [14]. Concerns regarding musculoskeletal toxicity in children are based on a study in juvenile animals showing the development of erosive arthropathy in weight-bearing joints [34]. Safety data on fluoroquinolones in humans are limited, but some clinical trial data suggest that adverse musculoskeletal events in children are usually mild and reversible [14, 34–36]. Our meta-analysis results provide information about the clinical efficacy of fluoroquinolones in achieving defervescence within 48 h of administration in pediatric patients with MRMP infection. Therefore, fluoroquinolones may be an appropriate option for the treatment of MRMP infection in children.

In adults, there are concerns regarding fluoroquinolone resistance to respiratory pathogens other than MP [37]*.* In addition, fluoroquinolones have induced resistance in MP strains in vitro [38]. Accordingly, although the guidelines of the Infectious Diseases Society of America and the American Thoracic Society do not include detailed recommendations for alternative antibiotic treatment for MRMP, macrolides or tetracyclines are recommended as a first-line treatment for adults with MP infection, and fluoroquinolones as a second-line therapy [39]. Because there are concerns that indiscriminate use of quinolones may delay tuberculosis diagnosis in countries with a high prevalence of tuberculosis [40], such as China and Korea, the use of quinolones should be limited only to patients diagnosed with MRMP infections.

Our study had several limitations. First, all included studies had a small number of patients and relatively heterogeneous methodologies. There were differences among studies in terms of the type, dose, and treatment duration of tetracyclines and fluoroquinolones used, MRMP diagnosis, and study protocol. Second, the quality of the studies included in the analysis varied substantially, which may have affected the robustness of our outcomes. To overcome these limitations, we evaluated the included studies using credible tools (Cochrane Risk of Bias Tools for RCTs and the revised Risk of Bias Tool for Non-Randomized Studies for observational studies) that could appropriately evaluate the risk of bias; the risk of bias assessment results is summarized in Figs. 2–7. In addition, a random-effects model was used to overcome the heterogeneity that can be caused by this bias, and subgroup analysis was performed in cases of heterogeneity. Third, as the studies included in our analysis did not provide sufficient data on the safety of tetracyclines or fluoroquinolones, a meta-analysis of safety was not possible. None of the studies that described safety reported cases that were affected by adverse effects. Fourth, in this meta-analysis, we only included tosufloxacin, which is an oral fluoroquinolone approved for administration to children with otitis media or pneumonia in Japan, in the fluoroquinolone group. However, there have been reports that other fluoroquinolones, such as levofloxacin, have also been used to treat MRMP infection in children [41, 42], but they were excluded because they did not meet the inclusion criteria of this study. Further comparative studies are needed to evaluate the efficacy of other fluoroquinolones to treat MRMP infection in children. Finally, as there were limited studies on the use of fluoroquinolones in children with MRMP infection, we could not compare the length of hospital stay or therapeutic efficacy between fluoroquinolones and macrolides.

## Conclusions

The results of this meta-analysis revealed the clinical efficacy of tetracyclines and fluoroquinolones against MRMP infection in children. Although based on limited evidence, our results suggest that tetracyclines and fluoroquinolones may be used as a second-line therapy in the treatment of pediatric MRMP infection.

These results should be carefully interpreted, however, as the number of studies included was small and the study methodologies used were heterogeneous. In addition, the safety of tetracyclines and fluoroquinolones in children has not yet been established, and the studies analyzed did not provide sufficient safety data. More prospective clinical studies with a larger number of patients are required to validate the effectiveness and safety of tetracyclines and fluoroquinolones against MRMP infection in children.

## Supplementary Information

**Additional file 1.** Search strategies for database searching.

## Data Availability

The datasets used and/or analyzed during the present study are available from the corresponding author (E-mail: khkim99@catholic.ac.kr) on reasonable request.

## Abbreviations

CAP: Community-acquired pneumonia
OR: Odds ratio
RCT: Randomized controlled trial
MP: *Mycoplasma pneumoniae* Pneumonia
MRMP: Macrolide-refractory *M. pneumoniae*
WMD: Weighted mean difference
